# A New Method of Treating Diphtheria

**Published:** 1885-10

**Authors:** R. J. Nunn

**Affiliations:** Savannah, Ga.


VOL. II. OCTOBER, 1885. NO. 8.

### Original Communications.

### A NEW METHOD OF TREATING DIPHTHERIA.

READ BEFORE THE MEDICAL ASSOCIATION OF GEORGIA AT ITS THIRTY-SIXTH ANNUAL SESSION, AT SAVANNAH, GA., APRIL 15, 1885. BY R. J. NUNN, M. D., SAVANNAH, GA.

In the study of a successful mode of treating diphtheria, two aims must be kept constantly before the mind: 1. To neutralize the constitutional taint. 2. To remove and arrest the formation of local deposits.

When the diphtheritic deposit takes place only in localities where vital processes are not interfered with, its removal may not be a matter of very great importance, but where, as in the supra-laryngeal deposit, suffocation is a near possibility, then it becomes a matter of prime importance that the spread of the pseudo-membrane should be controlled. The local treatment herein suggested is intended to be applied to deposits in supra-laryngeal mucous surfaces, but it is quite possible that with suitable changes in the mechanism of application the same agents might be useful in other localities.

It goes without saying that the principle of the constitutional medication is applicable not only in all forms of diphtheria, but in other cases of general disturbance dependent on a systemic microbic poisoning.

There is no intention in this paper to discuss the question of the local or of the constitutional origin of diphtheria. It is the safes plan to assume that both theories are right, and to combat on both avenues of infection.

As to the microbic origin of fermentations and of infectious diseases, there is now but little discussion. The day of disputation has passed; the question at issue now is, touching the identification of the microbe producing a particular disease.

A word as to the prevention of infectious diseases outside of what is known as sanitation. M. Pasteur's experiments have proved that one of the best microbic filters known is the healthy organism of the higher animals. While in a condition of health it permits no foreign germ to enter, and tolerates none so long as it has not succumbed to disease. Therefore, the first element of prevention is the maintenance of the body in the highest state of physical health. How best to accomplish this is a question to be discussed elsewhere.

Assuming that the cause of diphtheria is a microbe, the destruction of the germ may be accomplished in two ways:

1. By chemical re-agents, known as antiseptics, etc.

2. By the discovery of some other micro-organism, which will eat up or destroy the microbe of diphtheria.

This paper has to do with the chemical re-agent only.

Supposing that the microbe of diphtheria exists in the blood, let us stop a moment to inquire if we are in possession of an antiseptic which will prevent the germination of its spores. In this investigation we must at present rely to a great extent upon what seem to be necessary conclusions, rather than upon demonstrable facts.

Dr. S. Miguel has published a table showing the relative value of a number of antiseptics. "It gives the weight in grammes or fractions of grammes of each of these microbicides capable of rendering imputrescible one litre of beef tea." The table contains the names of forty-seven articles. The list is headed by biniodide of mercury, 0.025. Third upon the list is oxygenated water, 0.05. The 14th place is assigned to salicylic acid, 1.00. Iodide of potassium occupies the 42d place, 140.00.

Iodide of potassium can be given in such quantities, without discomfort to the patient, that it will appear in the urine in amount sufficient to prevent the development of bacteria and consequent decomposition. I have preserved specimens of such urine unchanged for months. To appear in the urine the salt must preexist in the blood, and if it can produce such results in one fluid, it is fair to conclude that it will produce similar effects in the other. According to Dr. Miguel's experiments, the proportion of iodide of potassium required to render the beef tea imputrescible was about 1-7th. Very much less than this proportion will produce a like result in the urine, as just mentioned, say from half an ounce to an ounce given daily to an adult, which, allowing a daily excretion of two quarts of urine, would give a possibility of only 1-128th to 1-64th, say nine times less than Dr. Miguel's proportion.

Following the table of Dr. Miguel again, it will be observed that the proportion of biniodide of mercury to beef tea would be as one to forty thousand; assuming a child to weigh fifty pounds or 35,000 grains, one grain would be sufficient to render the whole mass thoroughly aseptic, but if the results attained with iodide of potassium can be taken as a guide, this quantity may safely be divided by 9 or even by 18 so that the 1-18 of a grain of biniodide of mercury kept flowing through the system would thoroughly prevent the development of bacteria in a child weighing fifty pounds, but be it observed that even this minute proportion may be still farther diminished when it is administered in conjunction with iodide of potassium, which, as shown above, is in itself a powerful systemic germicide.

Theoretically, then, we may assume that a solution of biniodide of mercury in iodide of potassium, in the minute proportion just mentioned, is sufficient to prevent the development of the bacterial elements of disease. Upon this point it only remains to say that the theoretical anticipations just expressed were fully verified by practical results.

MODE OF ADMINISTERING THE SOLUTION.

It will be observed that one of the first indications to be fulfilled is to keep the system saturated with the antiseptic, and therefore the doses of the iodo-hydrargyrate solution must be given at short intervals. Iodide of potassium is said to have been observed in the urine six minutes after its administration; it is probable therefore that its elimination is rapid, which shows a greater necessity for frequent doses. Finally, the great value of the solution of biniodide as a local antiseptic is an imperative reason for frequently repeated doses. Of this, however, more anon.

STRENGTH OF THE SOLUTION.

The weakest solution I have used is one grain in eight ounces for very young children, but I usually employ a solution of one grain in four ounces. The proportion of iodide of potassium I have varied according to the age of the patient, the amount detected in the urine and the constitutional symptoms produced; upon this point, however, the experience of the physician must be the best guide.

LOCAL SYMPTOMS TO BE MET.

The deposit and spread of the pseudo-membranous deposit on the pharynx and the adjacent mucous membrane is a danger most imminent, calling for the promptest and most vigorous treatment. Presuming that a bacterium is a cause of the disease in question, frequent applications of any agents which may be employed to arrest the growth or destroy the bacteria are required. It has been proved by Cohn that the *bacterium termo* goes through its reproductive changes in an hour, and that this time may be greatly shortened by circumstances favorable to its development. It must not be forgotten that practically the bacterial multiplication is a continuous process, because wherever there are large quantities of bacteria there may always be found individual examples of each stage of development.

### PEROXIDE OF HYDROGEN.

The use of peroxide of hydrogen as a solvent of the membranous deposit of diphtheria has been elsewhere suggested by me, and I desire still to advocate its employment, because, while it is a most powerful solvent of the pseudo-membrane, it is without action upon the healthy mucous membrane, and it has the further advantage that in the dilute form to be had in the market it cannot be classed among the poisons, but I desire it to be understood that I am not wedded to this particular solvent, but that I consider a powerful dissolving agent as a most useful if not an absolutely necessary agent in the local treatment of diphtheria. With the peroxide of hydrogen the diseased membranes are thoroughly washed twice, thrice or oftener daily, according to the severity of the case, the washing being accomplished by means of the spray, the brush, the douche, or any or all means that the peculiarities of the case may require, or the ingenuity of the physician may suggest. There is, however, one drawback to the employment of this agent, viz.: that although rapidly dissolving and washing away recent deposits and pultaceous membrane, it seems almost powerless against thickened and hardened masses; hence has arisen the necessity for a

### SOFTENING OR DIGESTIVE AGENT.

Many applications have been suggested for this purpose, such as pepsin, pancreatin, trypsin, papayotin—all of these I have used, but it is to the last of these that I give the preference, because it acts equally well in solutions, having an acid or an alkaline reaction. I have not yet had an opportunity of trying the agavin, which is said to be a much more powerful digestive than any of the others.

The papayotin is applied to the membrane in powder by means of a powder blower, or it may be used in solution. The parts should first be thoroughly cleaned with the peroxide of hydrogen, and nothing should be given for twenty minutes or half an hour after the application, the object being to avoid washing off the papayotin before it has had time to act. I have been in the habit of making these applications twice or thrice daily.

OTHER LOCAL APPLICATIONS, which have been suggested, I have used ; among them the etherial solution of iodoform has proved most useful in my hands.

TO CARRY OUT THE TREATMENT.

1. Give five or six drops of the constitutional antiseptic in this case the solution of mercurial biniodide every ten minutes. If the patient is asleep the medicine may be dropped into the mouth with a "French pipette," or a camel's-hair brush, without awaking the child.

2. Twice or oftener daily, wash off all membrane in the throat, nasal passages, etc., with solution of peroxide of hydrogen or other suitable solvent.

3. Apply to the membrane papayotin or some agent possessing similar properties.

4. Let the patient alone for half an hour.

5. Resume internal medicine.

6. In the interval use etherial solution of iodoform, etc., locally.

7. Stimulants and nutriments are of course to be pushed as being the basis of all treatments.

8. As the case recovers, gradually the local applications are to be discontinued.

9. In like manner, the frequency of the doses of the constitutional antiseptic is to be lessened, but

10. It should not be wholly discontinued until all danger of sequels is past.

IN CONCLUSION.

It is not my intention to burden this paper with a long array of cases treated upon this plan, because there always occurs to the mind of the reader the possibility of errors of diagnosis. I will only say that the plan of treatment above proposed has been sufficiently successful in my hands to warrant me in bringing it before the profession and asking an extended trial, if not of the remedies mentioned, at least of the principles suggested, because it is quite possible that I may not have selected the very best medicines to fulfill the several indications.

The fact must be noted particularly, that the iodohydrargyrate solution is given in small quantities every few minutes. This is an important feature of the treatment. By this manœuver the mucous membrane of the mouth and pharynx is kept constantly bathed with the antiseptic solution, and no time left during which the tissue could be invaded by the bacterial growth. This is also, at the same time, probably, the best mode of administration for the purpose of keeping the system saturated with the germicedal fluid.

